# Multivessel Coronary Artery Vasospasm-Induced Takotsubo Cardiomyopathy

**DOI:** 10.1155/2022/2192863

**Published:** 2022-01-17

**Authors:** Vishal I. Patel, Serap Sobnosky

**Affiliations:** ^1^St. Mary Medical Center, Department of Internal Medicine, USA; ^2^UCLA David Geffen School of Medicine, USA; ^3^St. Mary Medical Center, Department of Cardiology, USA

## Abstract

Takotsubo cardiomyopathy is associated with a constellation of cardiac findings including reversible left ventricular dysfunction and an acute triggering stressor. Epicardial coronary vasospasm is a rarely reported etiology for takotsubo cardiomyopathy, and its pathophysiologic mechanisms still remain incompletely understood. We present the case of a 54-year-old female with chest pain and ST-elevation myocardial infarction who was found to have takotsubo cardiomyopathy due to diffuse multivessel coronary artery vasospasm in the absence of obstructive coronary artery disease. To our knowledge, this is the first angiographically confirmed case of this rare phenomenon to be reported, as most literature involves focal, segmental, or single coronary artery involvement. Moreover, we review current literature and develop a discussion on the targeted treatment of vasospastic disease as part of the multimodal approach to the management of takotsubo syndrome.

## 1. Introduction

Takotsubo cardiomyopathy, or takotsubo syndrome (TTS), is characterized by transient left ventricular dysfunction with apical dyskinesia, typically in the setting of a physical or emotional stressor. Initially thought to be a benign finding, further study over the past decade has revealed that TTS may be associated with severe complications including heart failure and sudden death, leading to intensification of understanding the underlying pathophysiological basis [1]. Of the several proposed mechanisms, multivessel epicardial coronary artery vasospasm is a rare and unique finding that is not yet well understood as most reported cases involve single arteries or focal vasospasm. Here, we present a case of TTS due to diffuse multivessel vasospasm of the right coronary artery (RCA), left anterior descending (LAD) artery, and left circumflex artery (LCx), managed with targeted vasodilatory and antianginal therapies.

## 2. Case Presentation

**Figure 1:**
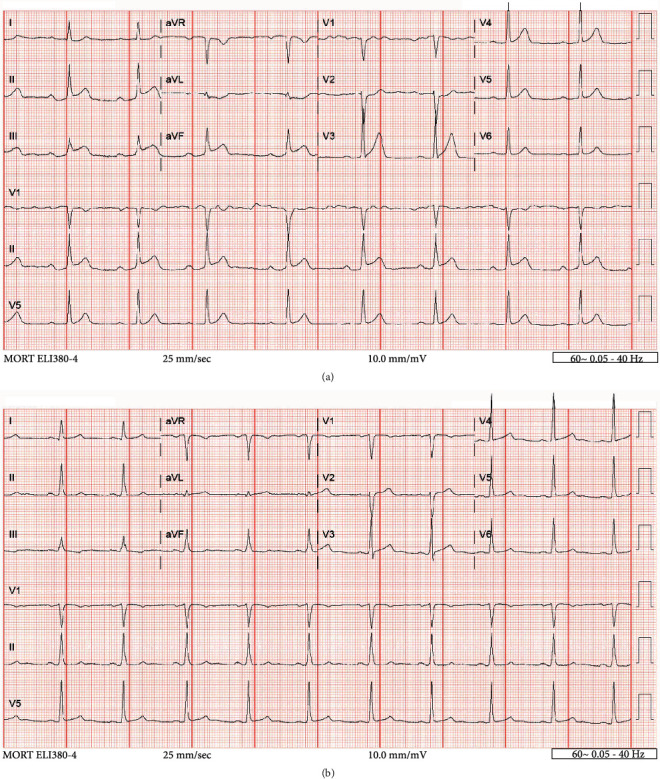
12-lead electrocardiogram. At presentation to the emergency department (a), showing sinus bradycardia (HR 51 bpm); 2 mm ST-segment elevations in leads II, III, and aVF with reciprocal depression in aVL; and first-degree AV block (PR 200 ms). Approximately one hour later (b), there is resolution of ST-segment changes in leads II, III, and aVF as well as resolution of first-degree AV block (PR 179 ms).

**Figure 2:**
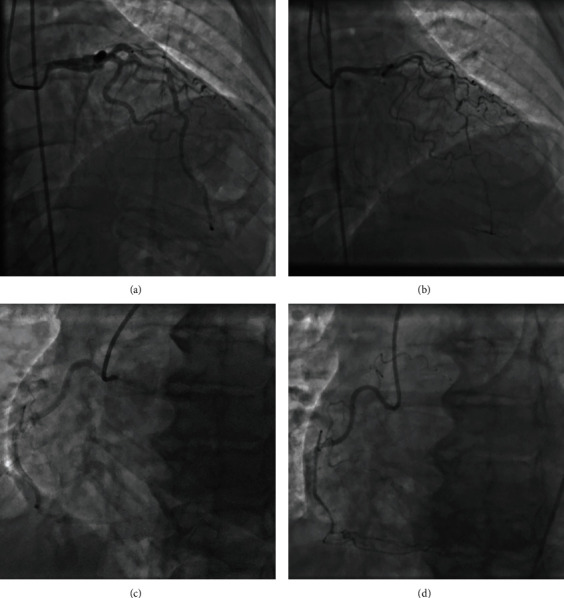
Coronary angiography demonstrates (a) normal left coronary artery system three years prior, (b) diffuse severe LAD and LCx vasospasm, (c) normal right coronary artery system three years prior, and (d) mild RCA vasospasm.

**Figure 3:**
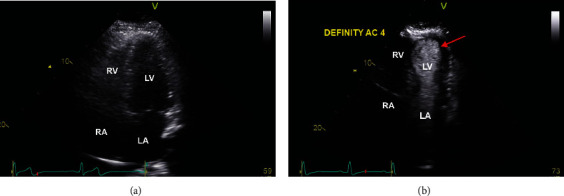
Transthoracic echocardiogram, apical 4-chamber view, demonstrates (a) normal LV contractility three years prior and (b) contrasted study with LV dysfunction with apical dyskinesia (red arrow), consistent with takotsubo cardiomyopathy.

A 54-year-old female was brought to the emergency department with nonexertional chest pain worsening for the past several hours. She reported profound psychosocial stress due to the recent sudden and tragic death of a grandchild. On examination, blood pressure was 188/112 mmHg with an otherwise unremarkable cardiopulmonary and vascular examination. Medical history was notable for hypertension, chronic obstructive pulmonary disease, tobacco dependence with a 30 pack-year history, peptic ulcer disease, and morbid obesity. Laboratory studies were notable for a troponin of 0.003 ng/mL and brain natriuretic peptide of 13 pg/mL. Electrocardiogram (ECG) revealed sinus bradycardia, first-degree atrioventricular block, and concave 2 mm ST-segment elevation in leads II, III, and aVF, with reciprocal depression in aVL (Figure 1(a)). Chest radiography demonstrated an enlarged cardiac silhouette but otherwise no acute pulmonary process. A computed tomography angiography of the chest was negative for aortic dissection. The patient was taken emergently to the cardiac catheterization laboratory for coronary angiography. A repeat ECG just prior to transport demonstrated sinus rhythm with essentially resolved ST-segment abnormalities seen on presentation (Figure 1(b)); however, the patient still had significant chest pain. Review of medical records, including coronary angiography three years prior, was notable for myocardial bridging of the left anterior descending (LAD) artery but no significant stenosis of the coronary arteries (Figures 2(a) and 2(c)). A transthoracic echocardiogram (TTE) three years prior had normal left ventricular (LV) contractility (Figure 3(a)).

In the cardiac catheterization laboratory, coronary angiography demonstrated diffuse vasospasm of the LAD which caused narrowing of the vessel diameter to 0.5 mm and reduced flow to thrombolysis in myocardial infarction (TIMI) 2 (Figure 2(b)). There was also vasospasm of the LCx as well as the smaller nondominant RCA (Figure 2(d)). There was no significant flow-limiting focal stenoses of the coronary arteries. A left ventriculogram also demonstrated apical ballooning; however, the images could not be retrieved due to a software error. The patient was given sublingual nitroglycerin 0.4 mg twice which resulted in rapid improvement in her chest pain and vasospasm. Further provocation testing was deemed unnecessary due to demonstrative real-time angiographic findings. A TTE with contrast revealed a moderately impaired LV systolic function with apical ballooning, associated with a depressed LV ejection fraction of 40% (Figure 3(b)) (Video 1). The clinical picture was consistent with takotsubo cardiomyopathy. She was initiated on medical management for vasospastic anginal disease with an oral calcium-channel blocker, long-acting nitrate, and low-dose beta-blocker. The final duration of these medications was to be determined on follow-up visits, with expected titration as symptoms improved. The patient's symptoms resolved, and she was discharged home in a stable condition. A repeat TTE was done 4 weeks later with resolution of apical ballooning and recovery of ventricular function, confirming the diagnosis (Video 2).

## 3. Discussion

Although described as early as the 1980s, the term “takotsubo cardiomyopathy” is credited to the work of Dote and Sato in the early 1990s based on their report of the characteristic LV appearance reminiscent of an “octopus trap” or “octopus pot.” Since then, the term “takotsubo syndrome” (TTS) has become the nomenclature recommended by expert consensus as it is more comprehensive; however, it is still commonly known as stress, ampulla, or takotsubo cardiomyopathy, broken-heart syndrome, or apical ballooning syndrome [1]. Based on recently revised diagnostic criteria, TTS is defined as a transient LV dysfunction in the absence of obstructive coronary artery disease (CAD), associated with new ECG changes or elevated cardiac biomarkers, and typically triggered by an emotional or physical stressor. TTS typically affects postmenopausal women, most commonly in the sixth decade of life [2]. Several pathophysiological mechanisms have been proposed, including myocardial ischemia, LV outflow tract obstruction, catecholamine cardiotoxicity, and autonomic nervous system modulation [3]. Many of these theories result from understanding the triggers or clinical settings in which TTS is diagnosed, including among others, sudden loss of a loved one, financial loss, surgery, trauma, drug intoxication, or intensive physical demands [2, 4]. Clinical presentation therefore can often be indistinguishable from acute coronary syndrome (ACS) [1, 2, 5]. As the incidence of TTS continues to uptrend annually, characterizing the underlying pathophysiology and targeting management remain key to impacting worldwide morbidity and mortality [3, 6].

Within the realm of myocardial ischemia as an etiological basis for TTS, data on epicardial coronary artery vasospasm remains rather sparse. Fairly few cases of angiographically confirmed vasospasm have been reported, and all have focal or single coronary artery involvement. Diffuse multivessel coronary artery vasospasm has not yet been reported at the time of our review of literature. Plausibility of the vasospastic theory has been studied through provocative testing to induce coronary vasospasm. Tsuchihashi et al. reported a retrospective analysis with 10 of 48 (21%) patients with TTS demonstrating positive provocative vasospasm, 5 of whom had both right and left coronary artery involvement [7]. Angelini reported a series of four cases of TTS in which provocative vasospasm could reproduce TTS echocardiographic findings or suggestive symptoms [8].

Given the rarity of coronary vasospasm in TTS, management typically defers to a case-by-case evaluation of clinical symptoms and management of potential triggers. Pharmacologic therapy of vasospastic disease is often the first step in intervention, with initiation of calcium-channel blockers, beta-blockers (in certain cases), and long-acting nitrates [4, 9, 10]. Further studies are needed to definitively characterize coronary vasospasm and the role of provocative testing in TTS, as well as optimization of medical therapies for the development of robust guidelines. We hypothesize that there may be a role for prophylactic intervention in high-risk scenarios to prevent vasospasm-induced myocardial injury. This case uniquely captured vasospastic disease in real-time linked to TTS in the appropriate clinicopathological setting, with demonstrated normal cardiac function and coronary angiography prior, as well as demonstrated resolution of LV dysfunction.

In conclusion, we presented the first angiographically confirmed case of diffuse multivessel epicardial coronary artery vasospasm-induced TTS in the setting of a known psychosocial stressor. This case highlights vasospastic disease as a culprit process and utilizes a targeted management plan to encourage further development of robust prevention and treatment strategies.

## Data Availability

No data were used to support this study.

## Abbreviations

ACS: Acute coronary syndrome
CAD: Coronary artery disease
ECG: Electrocardiogram
LAD: Left anterior descending artery
LCx: Left circumflex artery
LV: Left ventricular
RCA: Right coronary artery
TIMI: Thrombolysis in myocardial infarction
TTE: Transthoracic echocardiogram
TTS: Takotsubo syndrome.

## Supplementary Materials

Supplementary Materials Video 1: Transthoracic echocardiogram, apical 4-chamber view, with contrast, showing ventricular dysfunction with apical dyskinesia, consistent with takotsubo cardiomyopathy. Video 2: Transthoracic echocardiogram, apical 4-chamber view, showing complete recovery of ventricular function about 4 weeks later.
